# Stereotactic body radiation therapy in the treatment of oligometastatic prostate cancer

**DOI:** 10.3389/fonc.2012.00215

**Published:** 2013-01-22

**Authors:** Kamran A. Ahmed, Brandon M. Barney, Brian J. Davis, Sean S. Park, Eugene D. Kwon, Kenneth R. Olivier

**Affiliations:** ^1^Department of Radiation Oncology, Moffitt Cancer CenterTampa, FL, USA; ^2^Department of Radiation Oncology, Mayo ClinicRochester, MN, USA; ^3^Department of Urology, Mayo ClinicRochester, MN, USA

**Keywords:** stereotactic body radiation therapy, stereotactic radiosurgery, prostate metastases, intensity-modulated radiation therapy, prostate-specific antigen, bone metastases

## Abstract

**Purpose/objective(s):** To report outcomes and toxicity for patients with oligometastatic (≤5 lesions) prostate cancer (PCa) treated with stereotactic body radiation therapy (SBRT). **Materials/methods:** Seventeen men with 21 PCa lesions were treated with SBRT between February 2009 and November 2011. All patients had a detectable prostate-specific antigen (PSA) at the time of SBRT, and 11 patients (65%) had hormone-refractory (HR) disease. Treatment sites included bone (*n* = 19), lymph nodes (*n* = 1), and liver (*n* = 1). For patients with bone lesions, the median dose was 20 Gy (range, 8–24 Gy) in a single fraction (range, 1–3). All but two patients received some form of anti-androgen therapy after completing SBRT. **Results:** Local control (LC) was 100%, and the PSA nadir was undetectable in nine patients (53%). The first post-SBRT PSA was lower than pre-treatment levels in 15 patients (88%), and continued to decline or remain undetectable in 12 patients (71%) at a median follow-up of 6 months (range, 2–24 months). Median PSA measurements before SBRT and at last follow-up were 2.1 ng/dl (range, 0.13–36.4) and 0.17 ng/dl (range, <0.1–140), respectively. Six (55%) of the 11 patients with HR PCa achieved either undetectable or declining PSA at a median follow-up of 4.8 months (range, 2.2–6.0 months). Reported toxicities included one case each of grade 2 dyspnea and back pain, there were no cases of grade ≥3 toxicity following treatment. **Conclusion:** We report excellent LC with SBRT in oligometastatic PCa. More importantly, over half the patients achieved an undetectable PSA after SBRT. Further follow-up is necessary to assess the long-term impact of SBRT on LC, toxicity, PSA response, and clinical outcomes.

## INTRODUCTION

Prostate cancer (PCa) is the most common non-skin cancer in men in the United States, with 1 in 36 men dying from the disease (American Cancer Society, 2010). Androgen deprivation is often the treatment of choice for patients with a new diagnosis of metastatic or locally advanced PCa. In the metastatic setting, androgen deprivation is associated with a response rate of 80–90%, and overall survival estimates range from 24 to 36 months (Hellerstedt and Pienta, 2002). Patients with hormone-refractory prostate cancer (HRPC) often have distant metastatic disease, and bony metastases may result in significant morbidity and a decline in quality-of-life (Weinfurt et al., 2002). Current palliative treatment options for patients with hormone-refractory (HR) metastatic PCa include external beam radiation therapy (EBRT), and/or systemic radiopharmaceuticals, chemotherapy, bisphosphonates, surgery, and analgesics.

Stereotactic body radiation therapy (SBRT) uses similar techniques as central nervous system (CNS)-based stereotactic radiosurgery but treats tumors outside of the CNS using a coordinate system, which allows for limited but highly precise treatment fields. It offers some distinct advantages over conventional EBRT for patients with oligometastatic cancer, including a shorter treatment course, possibly better disease control (Yamada et al., 2008), and the ability to treat in the setting of re-irradiation while sparing more normal anatomy (Sahgal et al., 2009). In addition, SBRT offers a more effective radiobiologic dose (Fowler et al., 2004).

Patients with five or fewer metastatic lesions on diagnostic imaging studies are of a particular interest as their disease burden may be small enough that a change in a tumor marker, such as prostate-specific antigen (PSA) in PCa, may be noted and followed serially to monitor tumor response to treatment. In the current study, we report our center’s experience from a prospective database of patients treated with SBRT for oligometastatic PCa (≤5 PCa lesions on diagnostic imaging), many of whom had previously been diagnosed with HR disease.

## MATERIALS AND METHODS

This study included 17 patients with 21 oligometastatic PCa lesions treated with SBRT at our institution between February 2009 and November 2011. Data for these patients was prospectively collected and analyzed for this review. This study was approved by the Institutional Review Board (IRB) at our institution and informed consent was obtained from all patients. Eligibility included biopsy-proven PCa, Karnofsky performance status (KPS) >40 with life expectancy >3 months, and confirmation of metastases using (11)C-choline positron emission tomography/computed tomography (^11^CPET/CT; *n* = 7), magnetic resonance imaging (MRI; *n* = 6), biopsy (*n* = 1), CT (*n* = 1), or both ^11^CPET/CT and MRI (*n* = 2). Eleven patients had HRPC, which was defined as an increase in the serum PSA level over the baseline level ≥2 consecutive samples obtained ≥7 days apart despite receiving androgen deprivation therapy (ADT). In patients diagnosed with HRPC, a change in ADT sequencing from continuous to intermittent often occurred; however, ADT was not routinely discontinued or changed.

### SBRT TECHNIQUE

Techniques for SBRT immobilization and treatment planning from our center have been described previously and are summarized as follows (Macdonald et al., 2009). Each patient was immobilized using the Body-Fix whole-body or thoracic-T double vacuum immobilization system (Medical Intelligence, Schwabmünchen, Germany). Axial CT images were obtained on a Light Speed RT 16-slice CT simulator (GE HealthCare, Milwaukee, WI, USA). Image acquisition was set at 1.25-mm slice thickness. Four-dimensional CT images were obtained for patients treated to non-bony sites using the Varian Real-time Position Management (RPM) system (Varian, Palo Alto, CA, USA). Respiratory monitoring was conducted with an infrared reflector placed on the patient’s chest. Normal tissue and tumor segmentation was performed on the Advantage SimMD software (Advantage Workstation, GE Healthcare, Milwaukee, WI, USA). When available, diagnostic imaging (MRI, PET, contrasted CT scans) was fused to planning imaging to aid in gross tumor volume (GTV) delineation. For non-bony lesions, an internal target volume (ITV) was created from the GTV to account for tumor motion associated with patient respiration. The clinical target volume (CTV) was considered to be identical to the GTV for bony lesions or the ITV for non-bony lesions. The planning target volume (PTV) typically consisted of a uniform 5 mm expansion of the CTV. A dose–volume histogram was generated for organs in the vicinity of the PTV.

Both intensity-modulated radiation therapy (IMRT) and 3D-conformal SBRT treatment plans were designed using the Eclipse (Varian) treatment planning software. Often when IMRT was used, a heterogeneous dose distribution was achieved by prescribing a higher dose to the GTV relative to the PTV, creating a cloud of increased dose around gross disease while meeting radiation dose constraints on normal tissues adjacent or near the target volumes. Details regarding internally validated normal tissue organ-at-risk (OAR) constraints employed at our institution have been previously published (Barney et al., 2011).

Daily image guidance was performed using the ExacTRAC 6D X-ray system (Brainlab, Feldkirchen, Germany) with the 6D robotic couch for bony lesions or cone-beam CT (CBCT) for non-bony lesions. With ExacTRAC, initial corrections were automatically applied based on initial set-up imaging. Verification imaging using both tube-detector pairs was repeated to confirm positioning within 1 mm and 1°. Prior to delivering each treatment field, a “snap” verification image using a single tube-detector pair was acquired to verify positioning, and full imaging verification and shifting were repeated if the snap verification image was off by >2 mm. With CBCT, an initial CBCT scan was acquired, a match was manually performed, and the shift was applied. Any shift >0.3 cm required a second CBCT acquisition to verify position.

Stereotactic body radiation therapy dose was chosen based on previously published studies for the treatment of bony and soft tissue oligometastasis (Salama et al., 2008; Yamada et al., 2008), proximity of critical structures, and whether the area in question had been previously irradiated. The normal tissue constraints used for these treatments have been published previously (Barney et al., 2011; Ahmed et al., 2012) and are similar to those employed at other institutions with a high-volume SBRT practice.

### EVALUATION OF RESPONSE

Local control (LC) was determined by a patient’s most recent imaging and was defined as a lack of tumor progression within the PTV. Freedom from distant progression (FFDP) was defined as no development of new metastases and/or progression of untreated metastases. PSA testing was performed both immediately prior to SBRT and approximately 2 months after completion of SBRT. Follow-up thereafter included history and physical examination, serum PSA testing, and imaging studies as indicated every 2–3 months. Toxicity was evaluated and graded according to the National Cancer Institute (NCI) Common Terminology Criteria for Adverse Events (CTCAE) v3.0 (National Cancer Institute, 2006). Late effects were designated as events occurring 3 months following treatment.

### STATISTICS

The Kaplan–Meier (KM) method was used to estimate rates of LC, FFDP, and prostate cancer-specific survival (CSS). Survival and disease control were calculated from the end of SBRT. The Wilcoxon signed-rank test was used to test differences in pre-SBRT PSA values against 2-month post-SBRT values and at the time of most recent follow-up. In all cases, a *p*-value of <0.05 was considered significant. All statistical analysis was performed with JMP 8.0 (SAS Institute Inc., Cary, NC, USA).

## RESULTS

**FIGURE 1:**
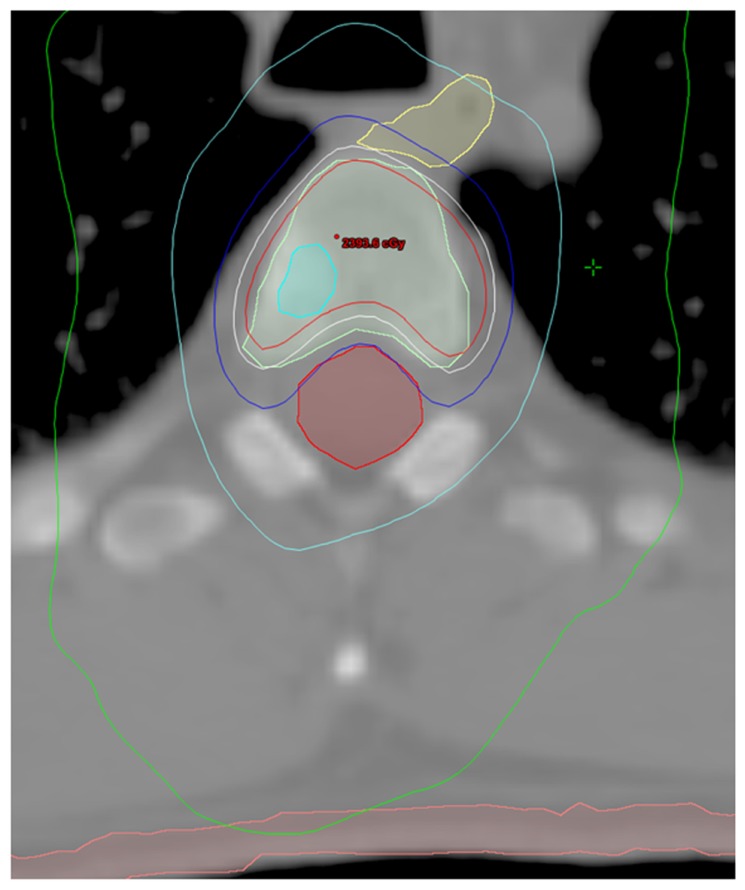
**Contours and isodose curves for a patient treated to 18 Gy in a single fraction to an right anterior T3 vertebral body metastasis.** GTV is shown in cyan. PTV is shown in light green. The spinal cord is shown in red. The esophagus is shown in yellow. The skin is shown in pink. The isodose curves are: red, 110% (19.8 Gy); white, 100% (18 Gy); blue, 80% (14.4 Gy); cyan, 50% (9 Gy); and green, 25% (4.5 Gy).

**Table 1** summarizes patient and disease characteristics at the time of SBRT. The median patient age for the cohort was 65.0 years (range, 50.6–79.7). Initial Gleason scores (GS) were as follows: GS 6 (*n* = 1), GS 7 (*n* = 9), GS 8 (*n* = 3), and GS 9 (*n* = 4). All patients had previously undergone definitive therapy prior to developing distant metastasis (DM). The median time from initial diagnosis of PCa to the development of DM was 50.4 months (range, 1.0–139.2 months). Eleven patients (65%) had HRPC at the time of SBRT, and in these men, the median duration of hormone therapy prior to developing HR disease was 14.0 months (range, 4.0–108.0 months). Likewise, the median duration from developing HR disease to treatment with SBRT was 13.0 months (range, 1.0–80.4 months). Sites treated with SBRT included bone (*n* = 19), lymph nodes (*n* = 1), and liver (*n* = 1). For patients with bone lesions, the median dose was 20 Gy (range, 8–24 Gy) in a single fraction (range, 1–3). **Figure 1** shows an example of target and normal tissue contours with associated isodose curves for a bony lesion located in the anterior portion of the T3 vertebral body. The patient with metastases to retroperitoneal lymph nodes was treated with 50 Gy in five fractions, and the patient with metastases to the liver received 60 Gy in three fractions. Fifteen patients (88%) received some form of androgen suppression after completion of SBRT.

**TABLE 1:** Patient and disease characteristics at the time of SBRT.

Characteristic	All patients, *n* = 17	HRPC, *n* = 11	Non-HRPC, *n* = 6
Lesions treated (*n*)	21	14	7
**Age (year)**			
Median	65.0	66.9	60.3
Range	50.6–79.7	52.9–79.7	50.6–74.0
**Initial Gleason score (*n*)**			
6	1	1	0
7	9	6	3
8	3	1	2
9	4	3	1
**Primary therapy at diagnosis (*n*)**			
Prostatectomy	15	9	6
EBRT	2	2	0
**Time from diagnosis to DM (month)**			
Median	50.4	63.0	28.0
Range	1.0–139.2	1.0–139.2	1.0–66.0
**Time to development of HRPC (month)**			
Median	–	14.0	–
Range	–	4.0–108.0	–
**Time from HRPC to SBRT (month)**			
Median	–	13.0	–
Range	–	1.0–80.4	–
**Site treated with SBRT (*n*)**			
Bone	19	12	7
Liver	1	1	–
Retroperitoneal	1	1	–
lymph nodes			

*HRPC, hormone-refractory prostate cancer; EBRT, external beam radiation therapy; DM, distant metastasis; SBRT, stereotactic body radiation therapy.*

**FIGURE 2:**
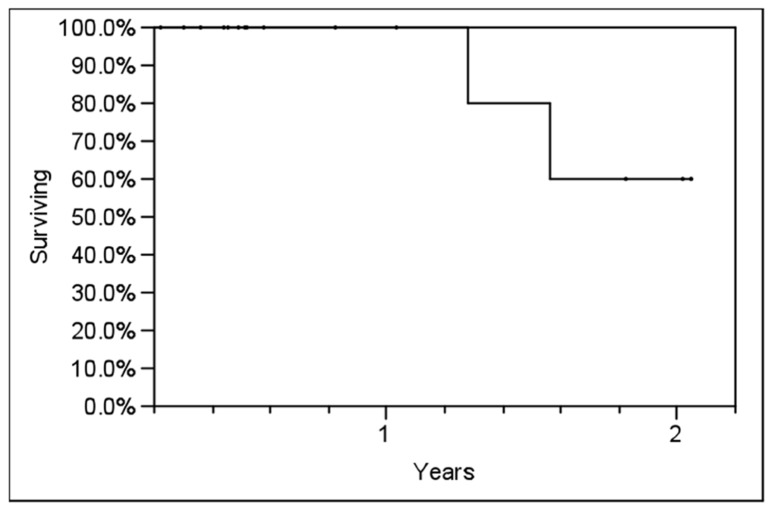
**Actuarial prostate cancer-specific survival (CSS) in all patients using the Kaplan–Meier method**.

**FIGURE 3:**
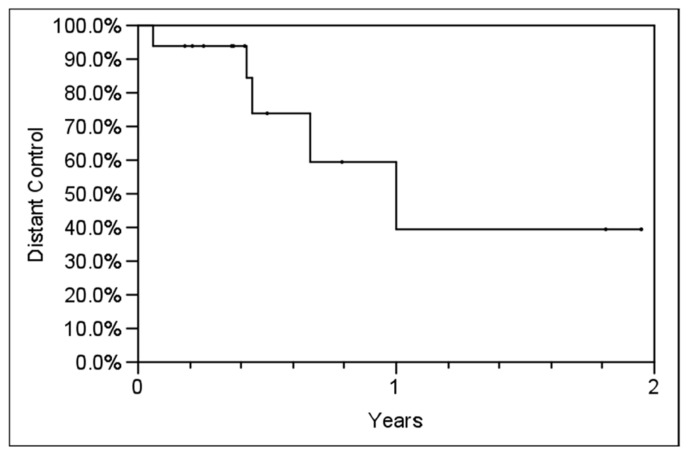
**Actuarial freedom from distant progression (FFDP) in all patients using the Kaplan–Meier method.** *Y*-axis represents probability of distant control.

**Table 2** outlines each individual patient’s treatment course and clinical outcome. Sixteen of the 21 lesions (76%) were evaluable for LC, and at a median follow-up of 6 months (range, 2–24 months), no patient had experienced a local recurrence. Six- and twelve-month estimates of CSS were 100% (**Figure 2**), though two patients had died from distant cancer progression at the date of last follow-up. Six- and twelve-month estimates of FFDP were 74 and 40%, respectively (**Figure 3**).

**Table 2:** Treatment outcomes for each patient.

Patient	GS	HRPC	Site treated	SBRT dose (Gy)/fractions	Post-SBRT HT	F/U time (month)	Initial PSA (ng/dl)	First post-SBRT PSA (ng/dl)	PSA at last F/U (ng/dl)	Acute toxicity	Grade
1	3 + 3	Y	L2	24/1	N	4.9	36.4	140	140	–	–
			L4	20/1							
2	4 + 5	Y	Rt 1st rib	18/1	N	5.3	17.9	14.2	1.7	–	–
			Lt scapula	18/1							
			L3–L5	18/1							
3	4 + 4	N	Rt sacrum	24/1	Y	2.2	2.7	<0.1	<0.1	–	–
4	3 + 4	Y	Lt ischium	24/3	Y	24.5	1.9	<0.1	69.4	–	–
5	4 + 3	Y	L4	24/1	Y	4.3	0.69	0.53	0.17	–	–
6	3 + 4	Y	RP LN	50/5	Y	4.4	1.1	<0.1	0.18	–	–
7	3 + 4	Y	Liver	60/3	Y	18.7	11.9	13.3	83	LFT elevation	1
8	4 + 5	Y	S1	18/1	Y	2.5	1.5	1.1	0.12	–	–
9	4 + 3	N	Rt sacrum	24/1	Y	4.9	5.2	1.1	<0.1	–	–
10	4 + 4	Y	Lt pelvis	24/1	Y	6.0	0.13	<0.1	<0.1	–	–
11	3 + 4	N	Lt 8th rib	18/1	Y	21.6	0.38	<0.1	<0.1	Dyspnea	2
12	4 + 4	N	T3	18/1	Y	23.4	2.1	<0.1	<0.1	Back pain/nausea	2/1
			T10	18/1							
13	4 + 5	N	L4	24/1	Y	9.5	3.7	0.59	<0.1	–	–
14	4 + 5	Y	L3	18/1	Y	5.3	4.9	4.7	1.2	–	–
15	3 + 4	Y	L5	24/1	Y	12.0	4.6	1.5	4.4	–	–
16	3 + 4	Y	Lt acetabulum	24/1	Y	2.2	1.4	0.58	0.46	–	–
17	3 + 4	N	Rt pelvis	30/3	Y	5.0	0.13	<0.1	<0.1	–	–

*GS, Gleason score; HRPC, hormone-refractory prostate cancer; SBRT, stereotactic body radiation therapy; HT, hormone therapy; F/U, follow-up; PSA, prostate-specific antigen; RP LN, retroperitoneal lymph nodes; LFT, liver function test.*

Prostate-specific antigen data was available for all 17 patients, and 9 (52.9%) patients reached undetectable PSA at a median of 3 months (range, 1–8 months) following SBRT. Of these nine patients, three (33%) had HRPC. With the exception of two patients (12%) who developed distant progression, all other patients (*n* = 15; 88%) experienced a decrease in PSA on the first post-SBRT measurement. In 12 patients (71%), the serum PSA had either yet to nadir or was undetectable at a median follow-up of 5.2 months (range, 2.2–23.4 months). In patients with HRPC, the PSA had either yet to nadir or was undetectable in 6 (55%) of the 11 patients at a median follow-up of 4.8 months (range, 2.2–6.0 months). The median initial post-SBRT PSA of 0.58 ng/dl (range, <0.1–140 ng/dl), was significantly lower than the median pre-SBRT PSA of 2.1 ng/dl (range, 0.13–36.4 ng/dl; *p* = 0.03). Similarly, the median PSA at the most recent follow-up (0.17 ng/dl; range, <0.1–140) was significantly lower than the pre-treatment PSA (*p* = 02).

No patient developed acute grade ≥3 toxicity following treatment. Reported toxicities included one case each of grade 2 dyspnea and back pain. The patient experiencing back pain also experienced grade 1 nausea. The patient treated for liver metastases experienced a transient increase in liver enzymes, alanine aminotransferase (ALT) and aspartate aminotransferase (AST), each grade 1. No late toxicity has been reported.

## DISCUSSION

Stereotactic body radiation therapy resulted in excellent LC in our cohort of oligometastatic PCa patients, the majority of whom had HR disease. More importantly, a quantifiable post-SBRT PSA response was observed in 15 of 17 patients, albeit in many patients who received hormone therapy post SBRT. PSA reached an undetectable level in nine patients, including three patients with HRPC. Likewise, 6 of 11 HR patients had either yet to experience a PSA nadir or had an undetectable PSA at the most recent follow-up. The long-term impact of this is unknown, but points to the biologic effectiveness of the SBRT in a HR setting.

Nearly two-thirds of patients with bone metastases will experience symptoms as a result of their bone disease (Coleman, 2001). Skeletal-related events (SREs), including pathologic fractures or spinal cord compression, are common in patients with HRPC, with 50% of patients with bone metastases experiencing ≥1 SRE (Lage et al., 2008). SREs are associated with decreased quality of life and increased health care expenditures (Lage et al., 2008). As such, patients with known bone metastases are followed closely for any signs or symptoms that could be indicative of progressive bone involvement. Potential interventions to prevent SREs include surgery, palliative EBRT, or other local therapies, such as cryotherapy and radiofrequency ablation. SBRT has a possible advantage in that it is both a non-invasive and highly tumoricidal treatment technique. Single fraction SBRT, which was used in the majority of the patients with bony lesions in the current study, has also been shown to be highly effective for the palliation of symptomatic bony lesions and the prevention of SREs (Yamada et al., 2008). In the setting of oligometastatic cancer, SBRT offers many advantages over conventional EBRT. With SBRT, patients are offered a shorter treatment course improving quality of life and time outside the hospital setting. Hypofractionated SBRT also offers a more effective radiobiologic dose, the ability to treat in the setting of reirradiation, and the ability to treat less normal anatomy (Mahadevan et al., 2011).

Monitoring tumor response to treatment is vital in the management of cancer patients. Unlike treatment in the definitive setting, where physical examination and imaging studies are required to determine treatment response, an improvement in the symptom(s) being palliated is often the most useful surrogate for treatment response in the palliative setting. Determining treatment response for patients with bony metastases in this setting can be difficult, as persistent tumor is often indistinguishable from post-treatment bone remodeling. For patients with bony metastases, a number of imaging-based algorithms have been set forth to evaluate tumor response to local therapy, including criteria from the World Health Organization (WHO, 1979), the International Union Against Cancer (UICC; Hayward et al., 1977), and MD Anderson (Costelloe et al., 2010). Yet the validity and reproducibility of some of these algorithms has been questioned (Hamaoka et al., 2010). For patients with metastatic PCa, serum PSA values, and PSA doubling time are frequently employed as metrics for the systemic burden of disease (Sandler et al., 2000; Smith et al., 2005). The findings of the current study, in which we were able to show sustainable changes in a quantifiable surrogate for tumor response such as serum PSA, are unique as this assessment can be made independently of imaging studies. One potential weakness of the study is that 15 of the 17 patients (88%) went on to receive additional hormone therapy after SBRT, including a majority of the patients with HR disease. Nonetheless, PSA appears to offer a minimally invasive and cost effective method of evaluating tumor response to treatment compared to serial imaging of the lesion in question.

With the advent of SBRT and other ablative local therapies, a more aggressive treatment approach is more often utilized for asymptomatic patients with oligometastatic disease (Okunieff et al., 2006; Salama et al., 2008). Recently, Muacevic et al. (2011) reported on the use of SBRT for bony metastatic PCa lesions. The study included 40 patients with 64 bone metastases, all treated with single-fraction SBRT. Patients were considered for SBRT regardless of their hormone therapy responsiveness. With a mean follow-up of 14 months, the authors report excellent response rates, with 6, 12, and 24-month estimates of LC of 95.5%. They also report significant decreases in PSA following treatment, with an initial median PSA of 5.4 ng/dl (CI: 1.4–8.2) that dropped to 2.7 ng/dl (CI: 0.14–10) 3 months following SBRT. In the current study, we report similar findings, with excellent LC and PSA response, albeit in a smaller cohort of patients. One unique finding of the current study is that over 50% of the patients with HR disease had either undetectable serum PSA levels or had PSA levels that were still declining.

Toxicity of treatment is an important consideration when treating patients with metastatic disease. SBRT used in this trial was well tolerated with only two reports of grade 2 toxicity in 21 treated lesions (9.5%) and no cases of grade ≥3 toxicity noted. This compares favorably with more traditional palliative regimens. For example, RTOG 9714 compared 8 Gy in 1 fraction to 30 Gy in 10 fractions (Hartsell et al., 2005). The grade 2+ toxicity rate was 17% for 30 Gy and 10% for 8 Gy. While this study is too small to accurately compare toxicity with large randomized studies, it does suggest SBRT regimens are very tolerable and perhaps more so than traditional palliative regimens.

Most other studies of SBRT for oligometastatic cancer, although retrospective in nature, have shown SBRT to be a favorable treatment modality due to a mild toxicity profile, improved disease control, shorter treatment course, and minimal invasiveness (Yamada et al., 2008; Sahgal et al., 2009; Stauder et al., 2011; Ahmed et al., 2012; National Cancer Institute, 2006). While intriguing, the results from our study should be validated in a in a prospective clinical trial. Doing so will allow for better identification of patients with metastatic PCa most likely to benefit from SBRT as opposed to other treatment options such as chemotherapy, immunotherapy, or less-aggressive localized palliative therapy such as conventional EBRT. It should also be noted that the majority of patients in this study received hormone therapy following SBRT, adding a confounding variable in our use of PSA as a measure of response to radiotherapy. Nonetheless, this study is one of few reports to assess SBRT for the treatment of PCa metastases that includes patients with HR disease.

## CONCLUSION

These preliminary results suggest SBRT is a safe and effective treatment for PCa metastases. A subset of patients with HR disease were found to have reductions in serum PSA values, with more than half of all patients achieving an undetectable serum PSA value. This study provides data suggesting SBRT results in excellent LC with a corresponding PSA response and an acceptable toxicity profile in properly selected patients with metastatic PCa.

## Conflict of Interest Statement

The authors declare that the research was conducted in the absence of any commercial or financial relationships that could be construed as a potential conflict of interest.
